# The Impact of Oral Contraceptive Initiation on Young Women’s Condom Use in 3 American Cities: Missed Opportunities for Intervention

**DOI:** 10.1371/journal.pone.0101804

**Published:** 2014-07-08

**Authors:** Chelsea Morroni, Stephen Heartwell, Sharon Edwards, Mimi Zieman, Carolyn Westhoff

**Affiliations:** 1 Institute for Women’s Health/Institute for Global Health, University College, London, United Kingdom; 2 Susan Thompson Buffet Foundation, Omaha, Nebraska, United States of America; 3 Department of Pediatrics, Mt. Sinai School of Medicine, New York, New York, United States of America; 4 Department of Gynecology and Obstetrics, Emory University, Atlanta, Georgia, United States of America; 5 Department of Obstetrics and Gynecology, Columbia University, New York, New York, United States of America; Johns Hopkins University Bloomberg School of Public Health, United States of America

## Abstract

**Purpose:**

To describe women’s condom use and assess predictors of consistent condom use and dual method use in the 6 months after the initiation of oral contraception (OC).

**Methods:**

We conducted a planned secondary cohort analysis among women less than 25 years of age initiating oral contraceptives at public family planning clinics in Atlanta, Dallas and New York City, USA, as part of a randomized trial. These clinics provide care to predominantly African American or Hispanic women of low socioeconomic status. Participants completed interviews at enrollment and at 6 months after OC start. We used multivariate logistic regression to assess factors associated with consistent condom and dual method use at 6 months.

**Results:**

1281 participants met the inclusion criteria for this analysis. At enrollment prior to OC start, 28% were consistent condom users. In the six months after initiation of oral contraception, only 14% always used a condom and 4% always used dual methods. In multivariate analysis, receiving basic advice to always use a condom after OC initiation from a provider during the baseline clinic consultation was associated with a 50% increase in the odds of using condoms consistently. Only 28% of participants were given this condom use advice.

**Conclusions:**

This study documents a decline in women’s condom consistent use subsequent to initiation of the oral contraceptive and suggests that opportunities for positive intervention around condom use among women starting hormonal methods are being missed. Basic condom use advice, which is neither time consuming nor resource dependent, was associated with increased consistent use and should be immediately implemented in all family planning services.

## Introduction

Oral contraception is one of the leading methods of contraception in the United States (U.S), used by 50% of contraceptors under 25 years of age. [1] Oral contraceptives (OC) have good contraceptive efficacy with typical use annual failure rates of 9%. [2] They also have the benefit of not requiring male involvement. However, like all contraceptive methods except for male and female condoms, OCs do not prevent sexually transmitted infections (STIs) and, as a result, women who use OCs may be at risk of STI if they do not also use condoms. In the U.S., young minority women are at greatest risk of all of the negative health outcomes associated with sexual activity, including unintended pregnancy [3], [4] and STI/HIV infection. [5], [6] Teenagers and young adults comprise more than half of reported gonorrhea and three-quarters of reported Chlamydia infections despite making up only 25% of the sexually active population. [6] Dual method use, defined as the use of a condom along with an effective non-barrier contraceptive (such as hormonal contraception, the IUD or sterilization) is the best option for those who require simultaneous protection from both pregnancy and STI, and a recent analysis using National Survey of Family Growth data shows that if users of highly effective contraceptive methods also used condoms, a substantial additional proportion of unplanned pregnancies and abortions could also be prevented [7].

Nationally representative survey data show that despite high rates of unplanned pregnancy and STI, U.S. women seldom combine condoms with highly effective contraceptives for optimal protection: estimates of dual use range from as low as 3–5% among Hispanic and African American youth [8] to 7.3–12% among all women of reproductive age [9], [10] to 14–20% in certain teenage populations. [11] While levels of dual use vary depending on the sample and the exact measure used, all estimates are low and underscore the need for better understanding and promotion of dual method use. *Healthy People 2020*, the national health objectives set out by the U.S. Department of Health and Human Services, recognizes the importance of dual method use and recommends that sexually active young people use condoms and hormonal contraceptives simultaneously to prevent pregnancy and STI [12].

Our understanding of factors associated with consistent dual method use is still relatively limited, and few studies have been designed to analyze continued condom use at the time that women initiate hormonal contraception. Most studies of the relationship between hormonal contraceptive initiation and condom use have been cross-sectional [7], [13]–[17], making inferences about the temporal relationship between hormonal method initiation and changes in condom use difficult, or they have been methodologically limited by small sample sizes or less stringent definitions of consistent condom and dual method use [13]–[20].

Most of the cross-sectional studies have documented an inverse relationship between use of long-term contraceptive methods, including oral contraception, and condom use [7], [15]–[17], and the scant prospective data generally show a decline in condom use among previously consistent condom users who initiate hormonal methods. [18]–[22] However, one prospective study has shown no decline in condom use among OC initiators after two years of follow-up. [19] Large, nationally representative, cross-sectional surveys [7], [9] and several smaller clinical studies [17], [19], [23], [24] have shown that OC users are more likely to simultaneously use condoms than users of other user-independent contraceptive methods such as the injectable, IUD and implant, but other studies have found no such association between contraceptive method type and condom use [15], [18], [25].

Given the ongoing predominance of OC use in the U.S. [1], [26], [27], and elsewhere in the world, a better understanding of the relationship between OC initiation and condom use is needed to help policy makers and health service providers identify strategies to sustain and increase condom use among OC initiators at ongoing risk of STI. And, given emerging concerns about the possible increased risk of HIV-acquisition with the use of some forms of hormonal contraception [28], [29] sustaining and increasing levels consistent condom use among hormonal contraceptive users is of great importance.

We report levels of condom use among young, minority women in the U.S. before and after initiation of oral contraception, examine how initiation of oral contraception influences condom use, and identify factors associated with consistent condom and dual method use after initiation of oral contraception.

## Methods

### Ethics statement

The Institutional Review Boards of Columbia University, Emory University, Mt. Sinai School of Medicine and University of Texas Southwestern approved this study. Participants provided written informed consent in their language of choice prior to enrollment.

### Study design

This cohort analysis addresses an *a priori* secondary aim of a randomized trial, the Quick Start Study, which compared the effect of two different approaches to OC initiation. [30] In brief, in the Quick Start Study women seeking OC were randomized to either immediate OC initiation at the time of study enrollment (Quick Start) or conventional OC initiation during their next period, and were followed-up for six months to determine 6-month OC continuation and pregnancy outcomes. Participants were recruited at publicly funded women’s health clinics in New York City, Dallas and Atlanta. These clinics provide care to predominantly African American or Hispanic women of low socioeconomic status.

After routine clinic consultation, clinic personnel referred women to the study if they were less than 25 years old, requesting OCs as their primary method of contraception, currently sexually active or anticipating becoming sexually active within 30 days of clinic visit, not currently using OCs, an implant, an IUD or a contraceptive injection not pregnant, and not desiring pregnancy within six months. Participants completed an in-person interview in Spanish or English to elicit socio-demographic, behavioral, sexual, reproductive and relationship characteristics, as well as contraceptive and condom use history. During the study, women received routine clinical care. Longer-term condom use counseling was provided during the routine clinic consultation according to clinic protocols and provider practice. Follow-up interviews were conducted by phone or in-person at six months (eligible visits could take place 25–32 weeks post-enrollment), and collected information regarding OC use, condom use, sexual activity, and pregnancy.

### Outcome measures

The outcome measures for this analysis were consistent condom use and consistent dual method use in the six months after OC initiation. Frequency of condom use was categorized for the six months before and after enrollment on a five-point condom use scale as: always, most of the time, some of the time, rarely or never. We collapsed these responses into two variables for most analyses: first, a three-level variable, defined as 1) always, 2) never or 3) inconsistent (most of the time, some of the time and rarely) condom use; second, a dichotomous variable defined as consistent (always) or inconsistent (most of the time, some of the time, rarely or never) use. OC use in the follow-up period was a dichotomous variable: continuous use (called ‘Continuing OC user’) vs. not (called ‘OC discontinuer’). OC discontinuation was defined as having not taken an OC pill in the last week. Consistent dual method use in the follow-up period was defined as follows: continuing OC use or use of another WHO Tier 1 or 2 effectiveness contraceptive method and always condom use.

### Data analysis

Analyses are restricted to the 1281 participants who had a follow-up interview, were sexually active both in the 6 months prior to and after enrollment, and for whom there is complete condom use data. Eighty percent of participants who were sexually active at enrollment completed the six-month interview and had sexual intercourse in the follow-up period (n = 1286). Complete condom use data before and after OC initiation was available on 1281 participants.

Data were analyzed using STATA version 11.0 (STATA Corporation, College Station Texas, USA). Bivariate associations were evaluated using student’s T-tests, chi-square tests, McNemar’s test (for non-independent observations in the pre- and post-OC initiation analyses), and Wilcoxon rank-sum tests. Variables that demonstrated significant bivariate associations (defined as p≤0.05) were entered into multiple logistic regression models to examine independent associations between participant characteristics and the outcomes of interest. Separate models were developed for 1) consistent condom use during the follow-up period and 2) consistent dual method use in the follow-up period. All models included study site. Variables were retained in the final logistic regression models if they demonstrated significant independent associations with the outcomes of interest and/or if their inclusion improved the model fit on likelihood ratio testing.

## Results

### Enrollment characteristics

Participants had a median age of 19 years, 94% were Hispanic or African American, 63% had completed less than a high school education, and one-quarter reported serious financial hardship (Table 1). Twenty percent had a history of STI diagnosis. Most had been pregnant at least once in the past (60%) and about one-fifth had previously had at least one abortion. At enrollment, 39% of participants (n = 492) reported condom use at last sexual intercourse, and 76% of these condom users (n = 376) reported that this was in part or mostly for STI prevention. Seventy-eight percent of participants (n = 999) reported the clinic provider told them to use condoms after starting OCs, but only 28% (n = 353) reported being counseled to always use condoms. Most of those who received any advice about condom use (n = 563, 56%) were advised only to use condoms as a back-up contraceptive in the first month of OC use. Women were significantly more likely to receive any advice to use condoms and advice to use condoms consistently if they were from Atlanta or New York, under the age of 18, African American, had a history of STI, had more than one sexual partner in the previous six months, and were not married or cohabiting with their partner. They were significantly less likely to receive any condom use advice if they had a history of consistent condom use (data not shown).

**Table 1:** Characteristics of sexually active young women initiating oral contraception.

Characteristic	N = 1281 n (%)
Median age (range); yrs	19 (12–24)
Completed high school/GED	469 (36.6)
Serious financial hardship in past 6 months	313 (24.5)
Race/ethnicity	
African American	436 (34.0)
Hispanic	774 (60.4)
Other	71 (5.5)
Number of sexual partners in past 6 months	
1 partner	1005 (78.5)
2 or more partners	276 (21.6)
Median length of sexual relationship with current partner (IQR); weeks	56 (20–144)
Relationship status among those with a partner	
Neither married nor living together	790 (61.7)
Living together, not married	282 (22.0)
Married	209 (16.3)
History of STI diagnosis	256 (20.2)
Given EC at enrollment clinic visit	176 (13.7)
Ever pregnant	753 (59.6)
Ever abortion, among all participants	230 (18.0)
Consistency of condom use in past 6 months	
Always	353 (28.0)
Most of the time	246 (19.2)
Some of the time	169 (13.1)
Rarely	99 (7.7)
Never	414 (32.0)
Condom used at last sexual intercourse	492 (38.5)
Main reason for condom use at last sexual intercourse	
Pregnancy prevention only	114 (23.1)
STI prevention	376 (76.2)

*Where values do not sum to 100%, observations are missing.

### Follow-up characteristics

The median number of weeks of follow-up was 30 (inter-quartile range: 28–31 weeks). Participants lost to follow-up were younger, more likely to be from Atlanta and New York, to have reported an STI history at enrollment, and to be recently post-partum. There were no differences in baseline condom use patterns among those who did and did not complete the six-month follow-up (data not shown). Thirty-two percent (n = 412) reported at least one new partner and 15% (n = 193) reported more than one sexual partner. Ninety-four women (7%) reported at least one new STI diagnosis. Forty-four percent (n = 566) were continuing OC users during the six-month follow-up period. In the 6 months prior to enrollment, 28% of women (n = 353/1281) always used condoms, 40% (n = 514/1281) used condoms inconsistently (most of the time, some of the time, or rarely) and 32% (n = 414/1281) never used condoms (Table 1). During the six month follow-up period after OC initiation, 14% of women (n = 183/1281) always used condoms, 71% (n = 913/1281) used condoms inconsistently (most of the time, some of the time, or rarely) and 14% (n = 185/1281) never used condoms.

### Influence of oral contraceptive initiation on condom use

Among the 353 women who reported consistent condom use in the six months prior to OC initiation, 48% (n = 170) stopped using condoms consistently after starting the OC (p<0.001). Thus, only half of previously consistent condom users continued consistent condom use after OC initiation (183/353 = 51%). Among the 414 sexually active participants who reported never using a condom in the six months prior to OC initiation, 30% (n = 125) never used a condom in the six months after OC initiation, 64% (n = 266) reported inconsistent use and just 6% (n = 23) became always users. Even among women who reported using condoms for STI protection prior to OC start (n = 376), condom use declined appreciably with most (56%) decreasing their level of condom use and only 28% using condoms consistently in the follow-up period (compared to 57% prior to OC start).

Fifty-six percent of women (n = 715) discontinued OC use in the follow-up period. Never use of condoms during follow-up was higher among continuing OC users than OC discontinuers (23% never use vs 8%, p<0.001). Of the 715 OC discontinuers, 81% (n = 579) continued sexual activity after stopping the OC. Of these 579 sexually active OC discontinuers, 248 (43%) were not using any method of contraception or condoms after OC discontinuation despite ongoing sexual activity; 12.6% (n = 73) had initiated another WHO tier 1 or 2 effectiveness contraceptive method, such as the IUD (n = 6), injectable (n = 31), patch (n = 33) or ring (n = 3), and 44% (n = 258) reported relying on condoms only after OC discontinuation, but at least 30% of these women, and likely a much greater proportion, were not using them at every act of sexual intercourse. Importantly, of the 353 women who were consistent condom users before starting the OC and who then discontinued the OC but continued to be sexually active (n = 233), at least 40%, and likely a greater proportion, did not resume consistent condom use after OC discontinuation, likely leaving them less protected from STI than they were prior to OC start. These above reported levels of inconsistent condom use (30% and 40%) represent a minimum possible level of inconsistent condom use after OC discontinuation. We do not have detailed data about consistency of condom use after OC discontinuation, but do have data on condom use at last sexual intercourse after OC discontinuation. If a woman reported no condom use at last sexual intercourse after stopping the OC, she could not have been a consistent condom user. But, it is likely that a proportion of women who did use a condom at last sexual intercourse did not always use a condom in the period after stopping the OC.

In bivariate analysis, women who reported consistent condom use in the 6 months after OC initiation were more likely to be from Atlanta, younger, and African American, and to have had more sexual partners, a shorter duration of current sexual relationship and to have had acquired a new partner in the follow-up period (Table 2). They were also more likely to have used condoms consistently prior to OC initiation, to have received advice to always use condoms at the time of OC initiation, and to not be continuing OC users. The study intervention (timing of OC initiation) was unrelated to condom use. In multivariate analysis, the predictors of consistent condom use remained similar (Table 2). Receiving advice from a provider during the clinic consultation to always use a condom after OC initiation was associated with a 50% increase in the odds of using condoms consistently. Consistent condom use was most strongly associated with acquisition of a new sexual partner in the follow-up period. The inverse relationship between consistent condom use and consistent OC use was maintained, though this association did not achieve statistical significance in the multivariate model. Neither race/ethnicity nor study site was associated with increased condom use in multivariate analysis.

**Table 2:** Characteristics associated with consistent condom use (n = 183) in the six-month period after initiation of oral contraception.

Characteristic of participant	Consistent condomuse, bivariate analysis		Consistent condomuse, multivariate analysis
	% (n)	p-value	Odds ratio and 95%confidence interval
Consistent OC user in follow-up period			
Yes	9.5 (51)	<0.001	0.6 (0.2; 1.1)
No	19.6 (132)		
Consistent condom user prior to OC initiation			
Yes	37.5 (91)	<0.001	2.9 (1.7; 4.9)
No	9.5 (92)		
History of STI diagnosis before OC initiation			
Yes	17.9 (43)	0.1	-
No	14.2 (136)		
Age at enrollment			
18+	10.1 (85)	<0.001	2.1 (1.2; 3.8)
<18	26.8 (98)		
Number of sexual partners in follow-up period			
1 partner	13.6 (137)	0.002	-
2 or more partners	22.7 (41)		
Race/ethnicity			
African American	26.0 (103)	<0.001	1.0
Hispanic	8.5 (64)		0.8 (0.4; 1.6)
Other	25.4 (16)		1.1 (0.4; 3.0)
New partner/main partner change in follow-up period			
Yes	26.6 (81)	<0.001	4.0 (1.2; 12.9)
No	10.7 (95)		
Length of current sexual relationship in follow-up period; weeks			
Median length among those who usedcondoms consistently, median (IQR)	46.1 (17.9; 100.8)	<0.001	-
Median length among those who didnot use condoms consistently, median (IQR)	87 (44.1; 172.8)		
Given advice to always use condoms			
Yes, always	23.9 (77)	<0.001	1.5 (1.1; 2.1)
No/not always	11.9 (106)		
Site			
Atlanta	27.8 (53)	<0.001	1.0
Dallas	7.1 (41)		1.7 (0.6; 4.9)
New York City	20.1 (89)		1.0 (0.5; 2.1)
Randomization group			
Immediate start	15.2 (95)	0.9	-
Conventional start	15.0 (88)		

Consistent dual method use, defined as continuous OC use or continuous use of another WHO tier 1 or 2 effectiveness contraceptive and condom use at every act of sexual intercourse in the six-month follow-up period, was 4% (51/1281). In bivariate analysis, consistent dual method users were younger, had used condoms consistently prior to OC initiation, were African American, had shorter sexual relationships, were given advice to always use condoms, and were from Atlanta (Table 3). Consistent dual method use was also very strongly associated with acquisition of a new sexual partner. As with condom use, neither race/ethnicity nor study site were associated with dual method use in multivariate analysis (Table 3).

**Table 3:** Characteristics associated with consistent dual method use (n = 51) in the six-month period after initiation of oral contraception.

Characteristic of participant	Consistent dual methoduse, bivariateanalysis		Consistent dualmethod, multivariateanalysis
	% (n)	p-value	Odds ratio and 95%confidence interval
Consistent condom user prior to OC initiation			
Yes	10.6 (27)	<0.001	-
No	2.4 (24)		
History of STI diagnosis before OC initiation			
Yes	3.5 (9)	0.8	-
No	4.1 (41)		
Age at enrollment			
18+	2.6 (23)	<0.001	2.2 (0.7; 6.9)
<18	7.3 (28)		
Number of sexual partners in follow-up period			
1 partner	3.7 (39)	0.2	-
2 or more partners	5.8 (11)		
Race/ethnicity			
African American	8.1 (34)	<0.001	1.0
Hispanic	1.4 (11)		3.0 (0.5; 17.0)
Other	9.0 (6)		1.4 (0.1; 15.1)
New partner/main partner change in follow-up period			
Yes	4.8 (17)	0.3	8.1 (1.7; 38.1)
No	3.5 (31)		
Length of current sexual relationship in follow-up period; weeks			
Median length among those who used condomsconsistently, median (IQR)	52.3 (21.0; 91.3)	0.07	-
Median length among those who did not use condomsconsistently, median (IQR)	79.4 (37.5; 158.6)		
Given advice to always use condoms			
Yes, always	6.5 (22)	0.008	-
No/not always	3.2 (29)		
Site			
Atlanta	10.9 (22)	<0.001	1.0
Dallas	2.2 (13)		0.2 (0.03; 1.9)
New York City	3.5 (16)		0.1 (0.02; 0.7)
Randomization group			
Immediate start	3.9 (25)	0.8	-
Conventional start	4.2 (26)		

## Discussion

Among young, mostly African American and Hispanic women of low socioeconomic status, this study documents a decline in consistent condom use subsequent to initiation of the oral contraceptive. In this STI high-risk population, almost half (48%) of women who used condoms consistently before initiating OC stopped consistent condom use, and almost no one increased levels of consistent condom use subsequent to OC initiation. Consistent dual method use was extremely low at 4%. Even among women who were using the condom for STI protection and who had risk factors for STI, consistent condom use declined appreciably. These levels of decline in consistent condom use parallel the findings of the few other prospective studies of this topic, which have found between a 25% and 50% reduction in condom use following initiation of the implant or injectable hormonal contraception [18], [20]–[22].

Dual method use of 4% in our population was considerably lower than some other recent studies, which have found dual use levels to be between 8 and 20%. [21], [26], [31]. These differences may be explained by differing study populations and our more stringent definition of dual use, which is the use of the OC or another WHO Tier 1 or 2 effectiveness contraceptive method plus the condom at *every* act of intercourse. Recent data from the national Youth Behaviour Risk Survey shows a small overall increase in dual method use among high school students from 6% in 1999 to 9% in 2009, with less dramatic increases among African American and Hispanic youth than Whites. Among African Americans, dual method use increased from 4.4% to 5.6% and among Hispanics it increased from 2.3 to 3.2%. [8] These national data are broadly consistent with our study findings of low dual method use, and suggest little progress with respect to dual pregnancy/STI protection among the most at-risk populations in the U.S. Factors associated with consistent condom use after OC initiation in our population were generally similar to those that have been described in other studies, including younger age, African-American ethnicity, previous condom use, shorter duration of sexual relationship and markers of STI risk [18]–[22], [32].

In our study, consistent condom use during the follow-up period was inversely related to OC continuation. While women initiating the OC may increase their protection against unintended pregnancy, they may place themselves at increased risk of STI. Thus, we may be seeing a trade-off between consistent condom use and OC use in this population, something that has been documented in other research. [33] However, condom use also declined appreciably among women who discontinued the OC. We were also able to demonstrate that a considerable proportion of women who used condoms consistently before starting the OC failed to resume consistent condom use after OC discontinuation, despite ongoing sexual activity. [22] Those OC discontinuers in whom condom use also declined may be at greater risk of both pregnancy and STI than they were before OC initiation [7], [21].

Several factors related to the observed changes in condom use have implications for family planning services and providers and may help to inform intervention strategies to increase condom use in this important population of young women. First, three-quarters of this sample received some advice to use condoms after starting OCs during their routine clinic consultation. However the content of the message varied considerably: only 28% were advised to always use a condom after OC initiation. The simple message to always use a condom, delivered by healthcare providers as part of routine care, was independently associated with consistent condom use in the follow-up period. Using our data, the number needed to treat (NNT), (which is the number of women starting OCs that would need to be counseled to use condoms consistently to prevent one women from inconsistent condom use,) for this simple and quick counseling intervention is 16. This means that about one in every 16 OC starters may benefit from this simple condom use message in terms of using condoms consistently in the 6 months after OC start. It is important to note that while condom use advice was independently associated with consistent condom use outcomes in our multivariate analysis, limited socio-demographic control variables were assessed, and we thus cannot fully exclude the possibility that certain women may have been both more likely to receive this advice and more likely to use condoms. However, a similar association between condom use messages and subsequent condom use has been observed in other prospective studies of hormonal contraceptive initiation, [20] and since providing this advice is cheap and fast, we believe our data supports the provision of condom use advice at OC initiation. Our data on condom use counseling was collected at the time of OC initiation, and the condom use data were collected at follow-up; thus, this association is unlikely to be subject to reporting bias.

Secondly, as may be expected, women who used condoms consistently before starting OCs were more likely to continue consistent condom use and/or combine the two methods during follow-up [18], [20], [22], but very few non-consistent condom users became consistent users. For many women, especially poor minority women, the only contact with preventive health services may be family planning visits and thus intervention with respect to condom use at these visits is essential. Failure to discuss future condom use represents a missed opportunity to help women decrease, or at least consider, their STI risk. Furthermore, substantial decreases in condom use occurred among the few women who were previously consistent users; however, in this study consistent condom users were much less likely to get any condom use advice at the clinic consultation than condom non-users (24% vs. 48%, p<0.001). Failing to counsel consistent condom users to continue this behavior while on OCs is missing an opportunity for a simple intervention that has a relatively high likelihood of success, given their prior success with condom use. This is particularly important for previous condom users who have known risk factors for STI or who used the condom previously at least in part for STI protection.

Thirdly, it is encouraging that women most likely to be at risk for STI were more likely to use condoms initially and to maintain condom use in the follow-up period, however a substantial proportion of women who did have a new partner (73%) or multiple partners (77%) in the follow-up period were not protected from STI by consistent condom use. Reducing, stopping or not initiating condom use at the time of OC start may be appropriate for lower-risk women [7], [34], [35]; however, monogamy on the part of the female partner does not ensure lack of STI risk/exposure in their male partners. It is encouraging that women perceived to be at higher risk for STIs by themselves and/or their health care provider were more likely to receive the message to use condoms, but the fact that all women did not receive such advice is problematic since it is well documented that young adults substantially underestimate their STI risk [36], [37].

This research furthers our understanding of condom use among OC users, but there are limitations. This study was not randomized with respect to condom counseling at OC initiation. Nonetheless, this non-randomized design allowed us to assess the occurrence, content and effect of condom use advice in a real care scenario in public sector family planning clinics. Secondly, we asked about level of condom use over a six month time period and dichotomized the variable into ‘always’ and ‘not always’ condom use. Due to our large sample size we were able to evaluate ‘always’ condom use on its own without combining this with ‘most of the time’; most other studies of this topic could not do because of insufficient sample sizes. [18]–[22] Because inconsistent condom (anything less than ‘always’ condom use) is much less effective in conferring STI protection than consistent condom use, our dichotomy is the most relevant outcome to assess with respect to the public health impact.

Unfortunately, we collected no sexual event-specific data such as casual or steady partner type, real or perceived STI/HIV risk, coercive or voluntary nature of sexual intercourse, and alcohol and drug use, but other studies have shown that event-level factors are highly predictive of condom use. [38], [39] Our indicators of STI risk were limited to history of STI, number of partners in the six months before enrollment, and new partner acquisition, number of new partners, and new STI diagnoses during follow-up. Thus, it is impossible to assess if the inconsistent condom users in this study were actually making good decisions and protecting themselves from STIs when a particular sexual event or partner put them at STI risk. Finally, the condom use outcomes in this study were self-reported. Thus, like most studies investigating the use of condoms, this study may be subject to reporting bias. Nonetheless, the women who reported consistent condom use were considerably less likely to report a new STI or pregnancy in the follow-up period than those who reported no or inconsistent condom use (STI: 2.3% vs. 7.8%, p = 0.01; pregnancy: 2.2% vs. 6.3%, p = 0.09), which points to the validity of our consistent condom use data.

## Conclusion

These data have implications for clinical care, public health, and future research. This study shows clearly that consistent condom use is low in this population before use of OC (only 28% were consistent condom users and about one-third never used condoms), that consistent condom use declines considerably once the OC is initiated, among both continuing OC users and OC discontinuers, and that a sizeable proportion of OC discontinuers fail to resume consistent condom use. These young sexually active African American and Hispanic women are thus at high and presumably continuing risk of STI, and clear opportunities for intervention are being missed by family planning services. Basic condom use advice, which is neither time consuming nor resource dependent, can, and should, be immediately implemented in all family planning services. Given STI-related morbidity and the possible increased risk of HIV-acquisition with some forms of hormonal contraception, particularly the progestin-only injectable [28], [29], makes the development and evaluation of interventions that focus on simple counseling messages and other low-resource interventions to increase consistent condom use, and thus dual protection, among hormonal contraceptive users a research priority. To date, there are few studies and little clinical evidence of effectiveness for interventions promoting dual method use and dual protection [40].
